# S100B Serum Levels in Schizophrenia Are Presumably Related to Visceral Obesity and Insulin Resistance

**DOI:** 10.1155/2010/480707

**Published:** 2010-06-10

**Authors:** Johann Steiner, Aye Mu Myint, Kolja Schiltz, Sabine Westphal, Hans-Gert Bernstein, Martin Walter, Matthias L. Schroeter, Markus J. Schwarz, Bernhard Bogerts

**Affiliations:** ^1^Department of Psychiatry, University of Magdeburg, Leipziger Str. 44, 39120 Magdeburg, Germany; ^2^Department of Psychiatry, University of Munich, Nußbaumstr. 7, 80336 Munich, Germany; ^3^Institute of Clinical Chemistry & Pathobiochemistry, University of Magdeburg, Leipziger Str. 44, 39120 Magdeburg, Germany; ^4^Max-Planck-Institute for Human Cognitive and Brain Sciences, P.O. Box 500355, 04103 Leipzig, Germany; ^5^Day Clinic of Cognitive Neurology, University of Leipzig, Liebigstr. 16, 04103 Leipzig, Germany

## Abstract

Elevated blood levels of S100B in schizophrenia have so far been mainly attributed to glial pathology, as S100B is produced by astro- and oligodendroglial cells and is thought to act as a neurotrophic factor with effects on synaptogenesis, dopaminergic and glutamatergic neutrotransmission. However, adipocytes are another important source of S100B since the concentration of S100B in adipose tissue is as high as in nervous tissue. Insulin is downregulating S100B in adipocytes, astrocyte cultures and rat brain. As reviewed in this paper, our recent studies suggest that overweight, visceral obesity, and peripheral/cerebral insulin resistance may be pivotal for at least part of the elevated S100B serum levels in schizophrenia. In the context of this recently identified framework of metabolic disturbances accompanying S100B elevation in schizophrenia, it rather has to be attributed to systemic alterations in glucose metabolism than to be considered a surrogate marker for astrocyte-specific pathologies.

## 1. Significance of S100B as Surrogate Marker for Glial or Blood-Brain Barrier Dysfunction in Schizophrenia

S100B is a secretory protein which is implicated in various intracellular and extracellular functional processes. Previous studies have indicated its involvement in the regulation of protein phosphorylation, cytoskeleton assembly, Ca^2+^homeostasis, transcription factors, and glucose metabolism [1]. Blood levels of S100B levels are increased in schizophrenia, as summarized in a recent meta-analysis of 13 studies involving 420 patients with schizophrenia and 393 control subjects [2]. Serum S100B reaches high effect sizes in schizophrenia compared to controls (mean ± SD: 2.02 ± 1.78), as confirmed by including only studies investigating drug-free patients (mean ± SD: 1.94 ± 1.33; *n* = 7). Elevated S100B levels were associated with acute exacerbations or deficit symptoms and have frequently been attributed to glial damage and dysfunction or blood-brain barrier leakage [2–5]. S100B is produced by astrocytes and oligodendrocytes and is acting as a neurotrophic factor with effects on synaptogenesis, dopaminergic and glutamatergic neutrotransmission [6–9]. Whitaker-Azmitia et al. observed a loss of dendrites in mice overexpressing S100B [10]. Interestingly, schizophrenia is indeed associated with certain haplotypes, leading to an increased S100B expression [11], and postmortem studies are suggestive of a progressive reduction of neuropil [12]. Therefore, a causal link between the aforementioned finding of elevated S100B levels and schizophrenia might be considered.

A recent analysis of cerebrospinal fluid (CSF) from first onset schizophrenia cases observed increased levels of S100B, without indications for impaired glial or neuronal cell integrity, as assessed by simultaneous measurement of non-secretory glial and neuronal proteins (glial fibrillary acidic protein/GFAP, myelin basic protein/MBP, neuron specific enolase/NSE) [13]. This finding speaks against the assumption of glial damage during acute psychosis, but could be interpreted as indirect evidence for an activated glial synthesis and release of S100B during acute psychosis. Of note, S100B has also been detected outside the nervous system, for example, in adipose and chondroid tissues, lymphocytes, melanocytes, the myocardium, and vascular endothelial/smooth muscle cells [8].

In conclusion, previous findings support the hypothesis that S100B is involved in the pathogenesis of schizophrenia, but elevated levels of this protein may not exclusively reflect brain- or glial-specific pathologies. Recently, Marchi et al. suggested that serum S100B is an ideal marker of blood-brain barrier integrity, because with a molecular weight of 21 kDa (S100B dimer) it may not penetrate through an intact blood-brain barrier [14]. Furthermore, its concentration is high in central nervous system fluids and low in blood. Indeed, serum levels of S100B were directly correlated with an experimental opening of the blood-brain barrier [14–16]. However, unlike in patients suffering from stroke, traumatic brain injury, or inflammatory brain disorders, there is no clear experimental evidence for a disruption of the blood-brain barrier in schizophrenia. Therefore, it remains unclear if serum levels of S100B are reflecting blood-brain barrier integrity also in schizophrenia.

## 2. Altered Peripheral and Cerebral Glucose Metabolism in Schizophrenia

Recent studies indicate novel interpretations of previous S100B findings in the context of disturbances in energy metabolism in schizophrenia (see below). Therefore, this section is briefly summarizing current knowledge on schizophrenia-related alterations in glucose metabolism.

Schizophrenia is characterized by a 20% higher mortality rate compared with the general population. Important contributing factors are an increased risk for type 2 diabetes and metabolic syndrome (defined by the American Heart Association as presence of three or more of the components: abdominal obesity, atherogenic dyslipidemia, elevated blood pressure, insulin resistance, prothrombotic state, and proinflammatory state). Weight gain and impaired glucose tolerance have been mainly attributed to side effects of atypical antipsychotic medication, such as clozapine and olanzapine [17–19]. However, impaired fasting glucose tolerance has also been reported in drug naïve schizophrenia cases [20–25] and unaffected siblings [26], suggesting disease-inherent abnormalities in peripheral glucose metabolism.

Cerebral insulin signaling seems to be likewise affected in schizophrenia [27, 28], probably causing disturbances in neural glucose uptake and utilization, as revealed by measurements of elevated CSF glucose levels [29], in vivo fluorodeoxyglucose positron emission tomography (FDG-PET) and functional magnetic resonance imaging (fMRI) studies [30–32]. State-dependent alterations in cerebral glucose-metabolism and functional disconnection have been found in brain regions which are involved in the pathophysiology of schizophrenia (e.g., of the prefrontal cortex, thalamus and mediotemporal lobe). The mediotemporal region and the hippocampus in particular appear to be of specific importance for cognitive impairment in schizophrenia. Studies addressing neuronal activation using fMRI have demonstrated that schizophrenic subjects show impaired patterns of hippocampal activity in novelty detection, declarative learning, and memory tasks [33–35]. Notably, it has been demonstrated that hippocampus-dependent memory performance can be improved by the administration of glucose in rodents and humans [36, 37]. In humans it has been shown that this effect is more pronounced when the task is cognitively demanding [37], or when the cognitive resources that can be applied for it are sparse, as is the case in the elderly and in schizophrenia patients [38, 39]. Of note, low rodent insulin-like growth factor 1 (IGF-1) concentrations were associated with impaired glucose metabolism in brain areas involved in learning and memory [40]. In schizophrenic subjects, clozapine has been shown to normalize disease-related IGF-1 deficits [41–43], and glucose administration boosts mediotemporal as well as dorsolateral prefrontal neuronal activity during the encoding into declarative memory [44, 45].

## 3. Adipose Tissue as a Potential Source of S100B

Adipocytes appear to be an important source of serum S100B since the concentration of S100B in adipose tissue is as high as in nervous tissue [46–49]. This fact has been barely considered during the past years. Remarkably, S100B is closely linked to the regulation of cellular energy metabolism. An immunoelectron-microscopic study suggested that S100B may be involved in the regulation of lipolysis [50]. The release of S100B from adipocytes is reduced by insulin, and activated by physiological factors such as stress (catecholamines and adrenocorticotropic hormone (ACTH)) or fasting [51–53]. Interestingly, a study on streptotocin-induced diabetes in Sprague-Dawley rats revealed a 2-fold increase in S100B protein levels in both brain and white fat tissue [54]. Therefore, an increased adipose tissue mass or changes of insulin metabolism such as insulin resistance most probably play a major role in increased S100B levels in schizophrenia too, given the increased prevalence of obesity and metabolic syndrome in patients and their first-degree relatives (see above) [55, 56].

## 4. S100B Serum Is Correlated with Body Mass Index and A-FABP in Healthy Human Subjects

**Figure 1:**
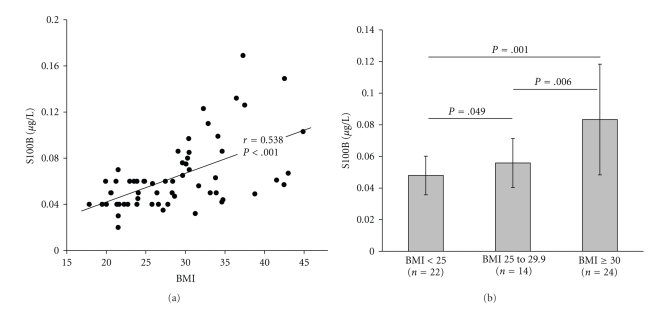
[57]: S100B serum levels were closely correlated with the BMI (a). Obese subjects (BMI ≥ 30) showed significantly elevated S100B levels in comparison to normal weight (BMI < 25) or overweight (BMI 25.0–29.9) subjects (b). *Annotation:* (a) *r* = Pearson correlation coefficient; (b) data are given as mean ± standard deviation (SD).

Indeed, a recent study showed a close correlation between body mass index (BMI) and serum S100B levels [57]. This study assessed S100B serum levels in 60 adult subjects (36 female, 24 male, age 22–58 years) with a BMI between 18–45 kg/m^2^ without a prior history of neuropsychiatric disorders. S100B levels correlated with the BMI (*r *= 0.538, *P* < .001), levels of leptin (*r *= 0.683, *P* < .001), and adipocyte-type fatty acid-binding protein (A-FABP; *r* = 0.801, *P* < .001) (Figure 1(a)). Accordingly, follow-up single group comparisons of BMI groups showed that S100B levels in obesity were significantly higher than in overweight (*P* = .006, Cohen's *d* = 2.25), and normal weight subjects (*P* = .001, Cohen's *d* = 2.90), or in overweight higher than in the latter (*P* = .049, Cohen's *d* = 0.65) (Figure 1(b)). A stepwise regression analysis showed that of the variables age, leptin, and A-FABP only the latter was significantly predicting S100B (*P* < .001). Correspondingly, new evidence from population studies and experimental animal models indicates that serum A-FABP is a powerful new risk marker for predicting metabolic syndrome and arteriosclerosis [58].

Effect sizes as measured by Cohen's d (see upper paragraph) indicated medium (0.5 < *d* < 0.8) to strong effects (0.9 < *d*) of BMI on S100B blood levels. This finding may explain previous reports indicating a direct relationship between S100B blood levels with body weight in anorexia nervosa: Effect sizes that were obtained from data given for anorexic subjects by Ehrlich et al. [59] before and after weight gain (i.e., mean ± SD of BMI: initially 14.5 ± 1.3, after >10% weight gain 17.1 ± 0.9; mean ± SD of S100B levels: initially 0.095 ± 0.041 *μ*g/l, finally: 0.128 ± 0.063 *μ*g/l) and by Holtkamp et al. [60] after 21 weeks of weight gain (mean ± SD of BMI: initially 14.8 ± 1.3, finally 17.0 ± 1.2; mean ± SD of S100B levels: initially 0.077 ± 0.023 *μ*g/l, finally 0.107 ± 0.035 *μ*g/l) were 0.81 and 1.3, respectively. In conclusion, S100B blood levels are directly related to BMI across an extensive range of nutritional states spanning from starvation to extreme obesity. Importantly, the effect sizes that BMI exerts on S100B blood levels in neuropsychiatrically healthy subjects (obesity compared to overweight: Cohen's *d* = 2.25; obesity compared to normal weight: Cohen's *d* = 2.90) are well within the range of effect sizes observed in schizophrenia (all studies: 2.02 ± 1.78; studies with drug-free patients: 1.94 ± 1.33) [2]. In conclusion, the correlation of S100B levels with other adipose-related measures, such as leptin and A-FABP, indicates that BMI or waist-to-hip ratio adjustments are strongly advised for future clinical studies examining peripheral blood S100B levels in order to avoid misinterpretation of results.

## 5. Elevated S100B Levels in Schizophrenia Are Presumably Associated with Visceral Obesity and Insulin Resistance

A recent study thus assessed S100B in both medicated and drug free schizophrenic patients along with the BMI, measures of glucose utilization and adipokine levels [61]. The subjects were comprised of 26 inpatients with acute paranoid schizophrenia and 32 control subjects, which did not differ significantly regarding age, gender, BMI and smoking habits (Table 1). Eleven patients were unmedicated for at least 6 weeks before admission (Table 2); 15 were already put on atypical antipsychotics (amisulpride, aripiprazole, clozapine, olanzapine, quetiapine, risperidone, or ziprasidone) for 26 ± 21 days, but still suffered from acute psychosis. Only benzodiazepines were allowed as additional psychotropic medication (for ≤6 days). Blood was collected between 9:00 and 11:00 AM (nonfasted). S100B and the following adipose-related factors were determined from serum samples: leptin, monocyte chemotactic protein 1 (MCP-1), hepatocyte growth factor (HGF), resistin, plasminogen activator inhibitor 1 (PAI-1), tumor necrosis factor alpha (TNF-*α*) and high-sensitivity C-reactive protein (hs-CRP). Levels of glucose, triglycerides and C-peptide^1^ were also assessed.

**Figure 2:**
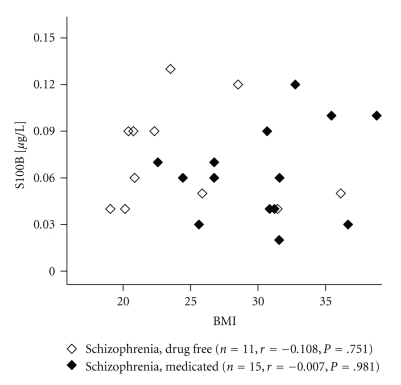
S100B levels in drug free and medicated schizophrenic patients were not correlated with the BMI. *Annotation: r* = correlation coefficient.

In control subjects, circulating S100B concentrations correlated significantly with the BMI (*r* = 0.540, *P* = .001), as in the abovementioned study in 60 subjects without a prior history of neuropsychiatric disorders [57]. Moreover, adipokines (leptin: *r* = 0.545, *P* = .001, HGF: *r* = 0.441, *P* = .012, resistin: *r* = 0.377, *P* = .033) and C-peptide/glucose ratios (an estimate of insulin resistance, *r* = 0.432, *P* = .014) predicted S100B levels. In contrast, circulating S100B levels in schizophrenia subjects were neither correlated with the BMI (drug free: *r* = −0.108, *P* = .751; with drug: *r* = −0.007, *P* = .981) nor with levels of leptin, HGF and resistin (Figure 2). It has to be clarified by future studies, if the different finding in schizophrenia may be explained by a predominant visceral fat distribution, which is not adequately assessed by the BMI. This idea is supported by our finding of MCP-1-correlated S100B levels in the patient group (*r* = 0.673, *P* = .023). MCP-1 is known to be particularly related to the visceral fat mass [62].

**Figure 3:**
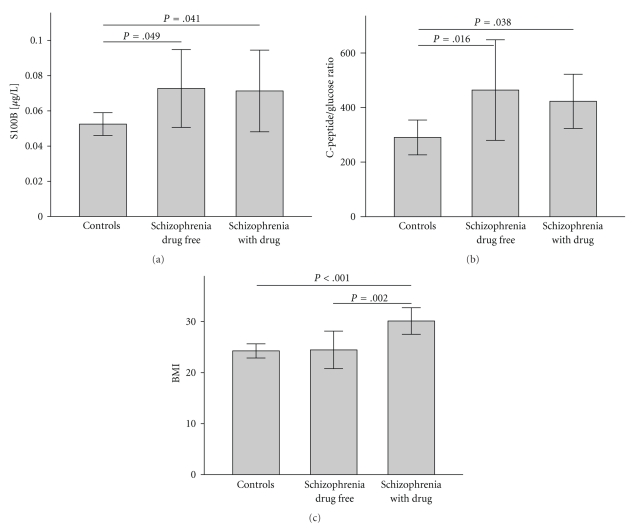
[61]: Elevated S100B serum concentrations (a) and an increased C-peptide/glucose-ratio (b), indicating insulin resistance, were schizophrenia-related. However, increases of BMI were primarily a consequence of antipsychotic medication (c). *Annotation: *Data are given as mean with 95% confidence intervals. Only *P*-values of significant group differences are displayed.

As illustrated in Figure 3 and Table 1, acutely ill schizophrenic subjects showed elevated S100B levels (*P* = .012). Indications of insulin resistance were revealed by increased glucose (*P* < .001), C-peptide levels (*P* = .002) and C-peptide/glucose ratios (*P* = .006). S100B and BMI were elevated in medicated schizophrenic patients (*P* = .041/*P* < .001), but controls with a BMI ≥ 25 were also found to show increased S100B levels (*P* = .025) and comparable correlations held true when adipokines were considered as predictors of S100B levels. A disease specific increase of S100B could however be demonstrated for closely BMI-matched drug free patients (*P* = .028, Table 2). Similarly, the finding of disease-related insulin insensitivity persisted when controlling for effects of medication, smoking or stress (ANCOVA).

These results are suggestive of insulin resistance in schizophrenia that may result in an increased release of S100B from adipose tissue. Commonly observed weight gain upon neuroleptic treatment would thus appear on the basis of an increased metabolic vulnerability in patients due to primary insulin resistance, which is also present independent of medication.

## 6. Studies on Insulin-Regulated S100B Release from Adipose Tissue Are Challenged by Findings in CSF and Brain Tissue

Schizophrenia-related disturbances in S100B expression are not specific for adipose tissue, but have also been observed in CSF and brain tissue (see above) [13, 63, 64]. Given the general distribution of energy consumption with the brain having the highest glucose turnover, especially under normal or resting conditions, a cerebral insulin resistance appears plausible, as opposed to a disease that primarily or exclusively affects peripheral adipose tissue [65, 66]. This hypothesis is in line with observations of a dysfunctional cerebral insulin receptor signaling in dorsolateral prefrontal cortex tissue from patients with schizophrenia [27]. Strikingly, insulin has been shown to downregulate S100B expression in astrocyte cultures and rat brain [54, 67, 68], in analogy to the abovementioned observations in adipose tissue [53, 69]. Previous cell culture experiments have shown that the expression and release of S100B from astrocytes and oligodendrocytes is stimulated by cellular glucose deprivation [6, 70]. A similar condition may occur in the brains of schizophrenia patients due to impaired glial glucose uptake as a consequence of insulin resistance.

Therefore, altered S100B protein expression in schizophrenia probably indicates systemic disturbances in cellular energy supply (e.g., by disrupted peripheral and cerebral insulin signaling) rather than adipocyte- or glia-specific pathologies. Upregulated insulin levels might represent a compensatory effort coming up against these insulin receptor disturbances. Indeed, a better psychopathology profile has been observed in acutely ill schizophrenic patients with higher insulin levels, potentially compensating these insulin receptor disturbances [71]. These considerations are offering promising new approaches in the therapy of schizophrenia-related metabolic and psychopathological alterations, for example the usage of glitazones, a group of insulin sensitizing drugs [72, 73].

## 7. Potential Relationship of S100B to the Metabolic Syndrome in Schizophrenia

According to the American Heart Association, the metabolic syndrome is characterized by a group of cardiovascular risk factors in one person (http://www.americanheart.org/ presenter.jhtml?identifier=4756). They include:

- *Abdominal obesity:* excessive fat tissue in and around the abdomen, elevated waist circumference: men—equal to or greater than 40 inches (102 cm); women—equal to or greater than 35 inches (88 cm).
- *Elevated blood pressure:* equal to or greater than 130/85 mm Hg.
- *Insulin resistance or glucose intolerance:* fasting glucose equal to or greater than 100 mg/dL.
- *Atherogenic dyslipidemia:* blood fat disorders—high triglycerides (greater than 150 mg/dL), low HDL cholesterol (men—less than 40 mg/dL; women—less than 50 mg/dL) and high LDL cholesterol—that foster plaque buildups in artery walls.
- *Prothrombotic state:* e.g., high fibrinogen or PAI-1 in the blood.
- *Proinflammatory state:* e.g., elevated CRP in the blood.

Interestingly, we previously observed an upregulation of the S100B/AGE scavenger sRAGE (soluble receptor of advanced glycation end products) during reconvalescence from acute schizophrenia [74]. Benefits of sRAGE have also been found in cardiovascular and metabolic disorders, which are associated with increased AGE levels, such as diabetes mellitus and arteriosclerosis [75, 76]. A closer look at the data from our recent study in Molecular Psychiatry reveals that some of the abovementioned metabolic risk factors were present in schizophrenia cases, but not all of them were linked to elevated S100B serum levels (Table 1) [61].

- *Abdominal Obesity:* We did not measure waist circumferences in this study, but blood concentrations of MCP-1 were elevated in the schizophrenia group (*P* = .034), indicating an increased visceral fat mass [62], and correlating with levels of S100B (*r* = 0.673, *P* = .023).
- *Elevated Blood Pressure:* This was an exclusion criterion of the study. Therefore, the relation of hypertension to S100B could not be analyzed.
- *Insulin Resistance or Glucose Intolerance:* Elevated glucose and C-peptide levels were observed in the schizophrenia cohort. C-peptide/glucose ratios predicted S100B levels (*r* = 0.432, *P* = .014).
- *Atherogenic Dyslipidemia:* Triglyceride levels were slightly elevated in schizophrenic subjects (*P* = .020), but were not correlated with S100B concentrations. HDL and LDL cholesterol were not systematically assessed.
- *Prothrombotic State:* Schizophrenia cases showed higher PAI-1 levels (*P* = .006) that were not correlated with S100B.
- *Proinflammatory State:* There was a trend towards elevated hs-CRP and TNF-*α* levels in schizophrenia. However, these inflammatory parameters were not related to S100B levels.

It remains unclear whether S100B may be considered as a predictor of metabolic syndrome in schizophrenia. This topic has to be elucidated by future studies in drug-naïve and prodromal schizophrenia cases with follow-up blood takes and clinical examinations in larger samples, comparing different standardized treatment regimens. Apart from the measurement of fasting glucose, triglyceride and HDL cholesterol levels in patients and controls, it will be necessary to assess additional clinical parameters such as blood pressure and waist circumference for a clear identification of patients at risk.

## 8. Summary and Conclusion

Several studies reported on elevated blood levels of S100B in schizophrenia, which have been attributed to glial pathology [2]. However, considerable amounts of S100B are released from adipose tissue during lipolysis. The release of S100B from adipocytes is reduced by insulin, and activated by physiological factors such as stress (catecholamines and adrenocorticotropic hormone) or fasting [46–48, 51–53]. Our recent observations in healthy human subjects are suggesting a close relation between serum S100B levels and the BMI, or levels of the adipose-derived factors leptin, HGF, resistin and A-FABP [57, 61]. Of note, previous studies showed that serum A-FABP is a powerful marker for predicting metabolic syndrome and arteriosclerosis [58].

Given the increased prevalence of visceral obesity and insulin resistance in schizophrenia, we recently analyzed the relation of serum S100B levels to the BMI and adipose derived hormones in acute paranoid schizophrenia [61]. The physiological relation of S100B to the BMI and the abovementioned adipose-derived factors was disrupted in schizophrenia. Several reasons may be hypothesized:

1. Schizophrenia-related disturbances in adipose tissue distribution, such as increased visceral fat which is better assessed by the waist-to-hip ratio instead of BMI. Interestingly, blood concentrations of MCP-1 were indeed elevated in the schizophrenia group, indicating an increased visceral fat mass [62], and correlated with levels of S100B (see above).
2. Schizophrenia-related disturbances in adipose tissue metabolism, such as an increased release of S100B, together with triglycerides and free fatty acids due to a predisposition to insulin resistance. Indeed, elevated glucose and C-peptide levels were observed in the schizophrenia cohort and C-peptide/glucose ratios predicted S100B levels (see above).
3. An increased release of S100B from brain tissue, as suggested by histological and CSF studies [13, 63, 64]. Notably, insulin has also been shown to downregulate S100B expression in astrocyte cultures and rat brain [54, 67, 68]. However, an animal study in Wistar rats suggests that the concentration of S100B may be differentially regulated in the periphery and the central nervous system: lipolysis following fasting was linked to an increased release of S100B to serum, while cerebrospinal fluid levels of S100B were not significantly altered [51]. This topic awaits further clarification.
4. Drug effects on cellular S100B production and release.
  - Glial cell culture experiments have shown that antipsychotic drugs can *directly* affect glial S100B release. Increased amounts of S100B were found in the supernatants of astroglial C6 cells treated with very high doses of risperidone [77]. In contrary, treatment of astroglial C6 and oligodendroglial OLN-93 cells with haloperidol and clozapine at a concentration corresponding to the assumed therapeutic dose range of these drugs reduced the release of S100B in vitro [78]. Other S100B-expressing cell types, like adipocytes, have not been tested yet in this context.
  - Alternatively, the potential influence of atypical antipsychotics on S100B via changing metabolic factors should be considered as a more *indirect mechanism*. Among the second-generation antipsychotics, clozapine and olanzapine are associated with the highest risk of weight gain, as well as changes in insulin sensitivity and lipid metabolism, which increase the risk of diabetes and cardiovascular disease [17–19].
5. Adrenaline, noradrenalin or ACTH-enhanced release of S100B from adipose tissue due to a schizophrenia-related activation of the endocrine stress axis [51–53]. This idea is supported by our finding of free cortisol index (FCI)^2^-correlated S100B levels in untreated acutely ill patients (unpublished results: *r* = 0.611, *P* = .046).

In conclusion, there is evidence for a novel link between S100B and disturbances of energy metabolism in schizophrenia, resulting in an increased release of S100B from brain and adipose tissue. Such systemic alterations in glucose metabolism may also affect glial S100B release. An upregulation of S100B may be a compensatory phenomenon, increasing intracellular energy supply by activating glycolysis (fructose-1,6-bisphosphate aldolase) and glycogenolysis (phosphoglucomutase) [79, 80]. Future studies in larger samples may focus on fasted drug naive schizophrenic subjects and the comparison of different standardized treatment regimens to further elucidate the suggested link between S100B and abnormal energy metabolism.

## Figures and Tables

**Table 1:** Demographics and clinical measurements of control subjects and patients with paranoid schizophrenia. *Annotation:* data are given as mean ± standard deviation (SD), n.a. = not applicable, ^a^ANOVA, ^b^Chi-square-test, ^c^ANCOVA, ↑↓ positive or negative influence on measures, bold *P*-values were significant, underlined *P*-values remained significant after Bonferroni correction.

Demographic data	Controls (*n* = 32)	Paranoid schizophrenia (*n* = 26)	*P-*values		
			Schizophrenia versus controls		
Age [*y*]	34.4 ± 10.8	34.7 ± 11.3	0.914^a^		
Duration of disease [*y*]	—	8 ± 9	n.a.		
Male/Female [*n*]	20/12	17/9	0.820^b^		
BMI [kg/m^2^]	24.3 ± 3.8	27.7 ± 5.7	0.196^a^		
Smokers/non-smokers [*n*]	14/18	16/10	0.178^b^		

Clinical data/measures	Mean ± SD	Mean ± SD	Influence of diagnosis (uncorrected ANOVA)	Influence of diagnosis (ANCOVA)	Influence of BMI (ANCOVA)
PANSS total score	—	84.8 ± 11.2	n.a.	n.a.	—
PANSS positive score	—	20.1 ± 4.9	n.a.	n.a.	—
PANSS negative score	—	22.1 ± 6.5	n.a.	n.a.	—
PANSS general score	—	42.7 ± 5.6	n.a.	n.a.	—

S100B [*μ*g/L]	0.052 ± 0.018	0.072 ± 0.038	**0.01**2^a^↑	**0.03**9^c^↑	0.384^c^

hs-CRP [mg/L]	1.76 ± 2.89	3.11 ± 2.68	0.073^a^↑	0.510^c^	$\underline{0.001^{c}↑}$
TNF-*α* [ng/L]	2.38 ± 1.62	3.45 ± 2.44	0.052^a^↑	0.193^c^	0.102^c^
Leptin [*μ*g/L]	9.62 ± 15.25	18.24 ± 22.96	0.093^a^↑	0.628^c^	$\underline{<0.001^{c}\;↑}$
MCP-1 [ng/L]	285.6 ± 155.3	397.0 ± 233.3	**0.03**4^a^↑	0.409^c^	$\underline{<0.001^{c}\;↑}$
HGF [*μ*g/L]	2.44 ± 1.92	3.83 ± 3.37	0.054^a^↑	0.488^c^	$\underline{<0.001^{c}\;↑}$
Resistin [*μ*g/L]	6.09 ± 2.11	6.31 ± 1.97	0.681^a^	0.893^c^	0.469^c^
PAI-1 [*μ*g/L]	1.47 ± 0.81	2.35 ± 1.50	**0.00**6^a^↑	**0.04**6^c^↑	**0.04**8^c^↑

Triglycerides [mg/dL]	146.1 ± 68.2	221.3 ± 160.1	**0.02**0^c^↑	0.088^c^↑	0.124^c^
Glucose [mg/dL]	82.9 ± 18.6	104.3 ± 21.6	$\underline{<0.001^{a}\;↑}$	$\underline{<0.001^{c}\;↑}$	0.894^c^
C-peptide [pmol/L]	1907.8 ± 1305.6	2572.8 ± 1376.5	$\underline{<0.001^{a}\;↑}$	$\underline{0.002^{c}\;↑}$	0.213^c^

**Table 2:** Demographics and clinical measurements of *drug free patients* with paranoid schizophrenia compared with *control subjects which were closely matched for BMI and smoking habits*. Annotation: data are given as mean ± standard deviation (SD), n.a. = not applicable, ^a^ANOVA, ^b^Chi-square-test, ↑↓ positive or negative influence on measures, bold *P*-values were significant.

Demographic data	Controls (*n* = 11)	Paranoid schizophrenia: drug free (*n* = 11)	Schizophrenia *versus* controls *P*-values
Age [*y*]	34.7 ± 12.0	35.1 ± 13.2	0.947^a^
Duration of disease [*y*]	—	8.5 ± 10.8	—
Male/Female [*n*]	7/4	7/4	1.000^b^
BMI [kg/m^2^]	23.5 ± 3.4	24.5 ± 5.5	0.621^a^
Smokers/non-smokers [*n*]	6/5	7/4	0.665^b^

Measures	Mean ± SD	Mean ± SD	
S100B [*μ*g/L]	0.046 ± 0.014	0.073 ± 0.033	**0.02**8^a^↑

hs-CRP [mg/L]	1.47 ± 1.64	2.50 ± 2.51	0.267^a^
TNF-*α* [ng/L]	2.51 ± 1.95	2.95 ± 1.79	0.584^a^
Leptin [*μ*g/L]	11.82 ± 16.91	13.74 ± 12.49	0.766^a^
MCP-1 [ng/L]	314.9 ± 190.0	308.8 ± 166.2	0.936^a^
HGF [*μ*g/L]	2.32 ± 1.85	2.84 ± 2.11	0.543^a^
Resistin [*μ*g/L]	5.71 ± 1.80	6.70 ± 1.83	0.214^a^
PAI-1 [*μ*g/L]	1.49 ± 0.75	2.30 ± 1.55	0.139^a^

Triglycerides [mg/dL]	144.3 ± 47.2	205.6 ± 102.3	0.093^a^↑
Glucose [mg/dL]	81.1 ± 12.2	105.7 ± 25.0	**0.01**1^a^↑
C-peptide [pmol/L]	1219.0 ± 680.1	2760.3 ± 1657.0	**0.01**3^a^↑

## Abbreviations

ACTH: Adrenocorticotropic hormone
A-FABP: Adipocyte-type fatty acid-binding protein
BMI: Body mass index
C6: An astroglial cell line from rat
CBG: Cortisol-binding globulin
CSF: Cerebrospinal fluid
FCI: Free cortisol index
FDG-PET: Fluorodeoxyglucose positron emission tomography
fMRI: Functional magnetic resonance imaging
GFAP: Glial fibrillary acidic protein
HDL: High-density lipoprotein
HGF: Hepatocyte growth factor
hs-CRP: High-sensitivity C-reactive protein
IGF-1: Insulin-like growth factor 1
LDL: Low-density lipoprotein
MBP: Myelin basic protein
MCP-1: Monocyte chemotactic protein 1
NSE: Neuron specific enolase
OLN-93: An oligodendrocytic cell line from rat
PAI-1: Plasminogen activator inhibitor 1
S100B: Member of a family of proteins that are 100% soluble in ammonium sulfate at neutral pH
SD: Standard deviation
sRAGE: Soluble receptor of advanced glycation end products
TNF-*α*: Tumor necrosis factor alpha.

## Endnotes

1. C-peptide is generated in the pancreas when proinsulin is split into insulin and C-peptide. It has no metabolic function. However, since C-peptide and insulin are secreted in equimolar amounts, the immunoassay of C-peptide permits the quantitation of insulin secretion. Moreover, C-peptide measurement has several advantages over immunoassays of insulin [81]: First of all, approximately 50% of the portal vein concentration of insulin is rapidly degraded by the liver whereas C-peptide extraction by the liver is only about 12%. As a result, peripheral venous levels of C-peptide more accurately reflect pancreatic insulin secretion than do peripheral insulin levels. Unlike insulin, which has significant uptake at target tissues, C-peptide is metabolically fairly inert. It is primarily removed from the circulation by the kidney, both by degradation and by urinary excretion. The half-life of C-peptide in the circulation is between two and five times longer than that of insulin. Therefore, C-peptide levels are a more stable indicator of insulin secretion than the more rapidly changing insulin levels. A very clear practical advantage of C-peptide measurement arising from its relative metabolic inertness as compared to insulin is that C-peptide levels in peripheral venous blood are about 5-6 times greater than insulin levels.
2. The FCI is a surrogate marker for free cortisol, which is the biologically active component. It is calculated by dividing the serum total cortisol [nmol/L] by the cortisol-binding globulin (CBG) level [mg/L].
